# Gadolinium‐Based Contrast Agents (GBCAs) for MRI: A Benefit–Risk Balance Analysis from a Chemical, Biomedical, and Environmental Point of View

**DOI:** 10.1002/gch2.202400269

**Published:** 2025-01-23

**Authors:** Angelo Scarciglia, Chiara Papi, Chiara Romiti, Andrea Leone, Enza Di Gregorio, Giuseppe Ferrauto

**Affiliations:** ^1^ Department of Molecular Biotechnologies and Health Sciences University of Torino Via Nizza 52 Torino 10126 Italy

**Keywords:** contrast agents, Gd(III), Gd‐based contrast agents, magnetic resonance imaging, pollution

## Abstract

Gadolinium‐based contrast agents (GBCAs) have revolutionized medical imaging, enhancing the accuracy and diagnostic value of magnetic resonance imaging (MRI). The increasing use of GBCAs has raised concerns about the release of gadolinium (Gd)(III) into the environment and potential risks for human health. Initially, multiple administrations of GBCAs were associated only with nephrogenic system fibrosis disease in individuals with impaired kidney function. Even if the Gd(III) retention in tissues has not yet been correlated with any specific disease, caution is required for the extensive use of GBCAs. The concerns related to the employment of GBCAs, due to the possible deposition and retention, should be extended also to healthy individuals without renal impairments. To ensure the well‐being of patients, there is a need to develop even more stable and better‐performing GBCAs, new MRI approaches requiring lower doses of GBCAs and, finally, innovative methods for recovering Gd(III) from both patients’ urines and the environment. This can have strong advantages for human health and for environmental sustainability, also considering Gd(III) scarcity, being a rare earth element, and the shared guideline to reduce, as much as possible, the use of rare metals.

## Graphical abstract

Gadolinium‐based contrast agents (GBCAs) revolutionize magnetic resonance imaging diagnostics but pose challenges due to their environmental impact and potential health risks. This article explores the chemistry, biomedical applications, and ecological concerns of GBCAs, proposing innovative strategies to mitigate these issues, including dose reduction, design of alternative contrast agents, and advanced recovery techniques.

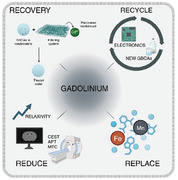

## 1 Introduction

### 1.1 Magnetic Resonance Imaging (MRI) and Gd(III)‐Based Contrast Agents (GBCAs)

Magnetic resonance imaging (MRI) is one of the most powerful techniques to perform diagnosis of different kinds of diseases, like cancer, inflammation, vascular pathologies, or neurodegenerative diseases. Moreover, it is used to assess the effect of applied treatments and monitor therapy outcomes.^[^ ^1^, ^2^ ^]^ It allows the visualization of soft deep tissues with great power in terms of spatial resolution and is one of the imaging technologies of the first choice at the clinical level since it does not employ ionizing radiation, thus ensuring good safety for the patients.^[^ ^3^ ^]^

Different factors are responsible for the MRI signal generation, which is affected both by instrumental parameters (magnetic strength, applied sequences, etc.) and by sample features such as proton density, endogenous presence of metals, longitudinal (*T*_1_) and transverse (*T*_2_) water protons relaxation times, etc. These last parameters strongly depend on the physicochemical properties of the microenvironment where water molecules are distributed and on the relationship between water and other molecules (e.g., proteins) or cellular components (e.g., biomembranes).^[^ ^4^, ^5^ ^]^ The differences in relaxation times among healthy and diseased tissues are at the basis of the generation of contrast (i.e., a difference in signal intensity) in MR images as it is well known that *T*_1_ of the tumor is intrinsically longer than *T*_1_ of the healthy tissues.^[^ ^6^ ^]^

MRI contrast agents (CAs) are chemicals able to reach diseased tissues and to modify *T*_1_ and *T*_2_ water relaxation times, so affecting the MRI signal intensity associated with the pathological tissue. This allows to discriminate the diseased tissues from the healthy ones (contrast generation and enhancement).^[^ ^7^, ^8^, ^9^, ^10^ ^]^ They contain paramagnetic metal centers with unpaired electrons that influence water spins relaxation. Clinical CAs contain Gd(III) ion, a rare earth element (REE)^[^ ^11^ ^]^ able to modify the relaxation times (especially *T*_1_) of water protons in regions where it distributes, thus generating a positive contrast (brightening) in *T*_1‐weighted_ MR images.

### 1.2 Gd(III)‐Based Contrast Agents and Applications in Clinical Practice

Gd(III) is a paramagnetic metal of REE family that has been used as a CA in MRI since the 1980s with the first Food and Drug Administration (FDA)‐approved molecule. The first GBCAs were introduced in the late 1980s and early 1990s, and they revolutionized the field of medical imaging by enabling the visualization of tissues and organs in detail.^[^ ^12^, ^13^ ^]^ These agents work by shortening the longitudinal relaxation time *T*_1_ of water molecules in the tissue, which results in an increase in signal intensity and improved positive contrast on *T*_1‐weighted_ MR images.

This allows for the detection of fine changes in tissue structure and function, making it easier for physicians to diagnose and, consequently, treat a wide range of conditions, including cancer, cardiovascular disease, neurological disorders, metabolic, inflammatory, and orthopedic disorders.

Over the years, various GBCAs have been developed, each with specific applications.^[^ ^14^ ^]^ For example, some agents are optimized for blood pool imaging,^[^ ^15^, ^16^ ^]^ while others are designed for imaging of the liver,^[^ ^17^, ^18^ ^]^ kidneys,^[^ ^19^ ^]^ or other organs. Today, GBCAs are widely used in clinical practice and have become an indispensable tool for the diagnosis and management of numerous diseases. Gd(III)‐contrast‐enhanced MR images are beneficial in the visualization of the edges of the tumor lesion, as well as for the detection of metastases, the evaluation of perfusion, and the staging of multiple sclerosis diseases.

**Figure 1:**
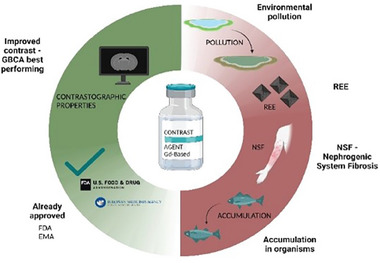
Benefits and risks balance of Gd‐based contrast agent use.

For this reason, GBCAs have been extensively tested for safety and efficacy and are generally well‐tolerated by patients. Despite this, some concerns about the potential toxicity of Gd(III) have been raised, and the evaluation of its accumulation in the body has become a topic of interest in recent years for biomedical and chemical scientists (**Figure** **1**).^[^ ^20^ ^]^

GBCAs are known to be the best‐performing MRI CAs since Gd(III) has seven unpaired electrons in its 4f‐orbital, which provide strong paramagnetic properties. Furthermore, Gd(III) displays a high magnetic moment (*µ* ^eff^ = 7.94), and long electronic relaxation time (*T*_1e_, *T*_2e_ = 10^−8^–10^−9^ s).^[^ ^8^, ^10^, ^21^, ^22^ ^]^ The dipolar interaction between the electromagnetic moment of the paramagnetic ion and the magnetic moment of water protons is indeed able to accelerate the relaxation rate of water spins, thus generating contrast. For all these properties, GBCAs can significantly shorten the *T*_1_ of water protons, inducing a brightening in *T*_1‐weighted_ MRI scans when the GBCA concentration in the site is enough (µm–mm range).

However, Gd(III) cannot be administered as a free ion because of its toxicity (LD_50_ is ≈0.1 mmol kg^−1^).^[^ ^23^ ^]^ A strong similarity among the ionic radius of trivalent Gd(III) and the bivalent Ca(II) is present, especially for the typical coordination numbers (CN) own by these metals in aqueous environment, coordinated by water (CN of 8–9 for Gd(III) and of 7–8 for Ca(II), with ionic radii ≈0.1 nm).^[^ ^20^ ^]^ This leads to a competition between Gd(III) and Ca(II) in several biological pathways where Ca(II) is normally present.

Already in 1989 Yang and Sachs, described how Gd(III) was able to block stretch‐activated ion channels in *Xenopus oocytes*.^[^ ^24^ ^]^

Thus, to prevent undesirable toxicity issues, and allow for a safer in vivo application, the Gd(III) ions must be bound in thermodynamically and kinetically stable complexes. Accordingly, different types of ligands have been designed with several structural and physicochemical features, thus obtaining suitable GBCAs.^[^ ^7^, ^10^, ^21^, ^22^, ^25^ ^]^

**Figure 2:**
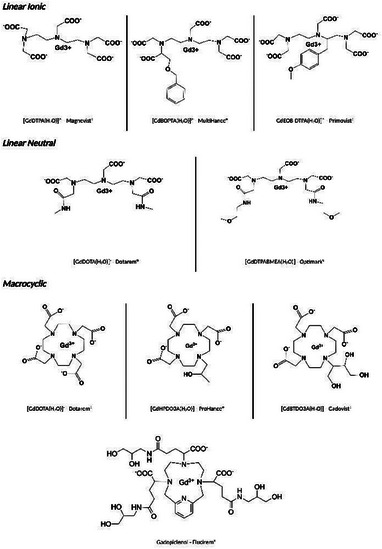
Chemical structures of clinically approved GBCAs.

Clinically available MRI GBCAs are linear or macrocyclic Gd(III) complexes, generally endowed with high thermodynamic and kinetic stability constants. The chemical structures of GBCAs approved for clinical use are reported in **Figure** **2**.

Very recently, gadopiclenol has been added to the series of clinical GBCAs showing improved relaxometric properties, in comparison to the other available CAs.^[^ ^26^, ^27^, ^28^ ^]^ The *r*_1p_ (i.e., longitudinal relaxation rate for 1 mm of GBCA) is ≈12.2 s^−1^ mm ^−1^ for gadopiclenol at 37 °C B_0_ = 1.5 T, in water this value is *≈*3–4 times higher than the one reported for the other clinical GBCAs.^[^ ^27^, ^29^, ^30^ ^]^ The comparison among the properties of the clinical GBCAs is reported in **Table** **1**.

**Table 1:** Main properties of some clinically approved GBCAs.^[^ ^27^ ^]^

International nonproprietary name	Gadopiclenol	Gadoterate	Gadobutrol	Gadoteridol	Gadobenate	Gadodiamide	Gadopentate
Trade Name	Elucirem	Dotarem	Gadovist	ProHance	MultiHance	Omniscan	Magnevist
Osmolality at 37 °C (mOsm kg^−1^ H_2_O) at marketed concentration	843	1350	1603	630	1970	789	1960
Log *P* (octanol/PBS for gadopiclenol, Butanol/H_2_O for others)	−4.2	−2.87	−2	−1.98	−2.33	−2.13	−3.16
Viscosity at 37 °C (mPa s) (0.5 m except gadobutrol at 1.0 m)	7.6	2	4.96	1.3	5.3	1.4	2.9
Relaxivity (*r*_1_/*r*_2_) mm ^−1^ s^−1^ at 37 °C							
0.47T In water In biological medium	12.5/14.6 13.2/15.1	3.4/4.1 4.3/5.5	3.7/5.1 6.1/7.4	3.1/3.7 4.8/6.1	4.2/4.8 9.2/12.9	3.5/3.8 4.4/4.6	3.4/4.0 3.8/4.1
1.5T In water In biological medium	12.2/15 12.8/15.1	2.9/3.2 3.6/4.3	3.3/3.9 5.2/6.1	2.9/3.2 4.1/5	4/4.3 6.3/8.7	3.3/3.6 4.3/5.2	3.3/3.9 4.1/4.6
3T In water In biological medium	11.3/13.5 11.6/14.7	2.8/3.3 3.5/4.9	3.2/3.9 5/7.1	2.8/3.4 3.7/5.7	4/4.7 5.5/11.0	3.2/3.8 4/5.6	3.1/3.7 3.7/5.2
Log K_Therm_	18.7	25.6	21.8	23.8	22.6	16.9	22.1
Log K_cond_ (pH 7.4)	15.5	19.3	14.7	17.1	18.4	14.9	17.7
Kinetic stability in acidic conditions (HCl, pH 1.2) and 37 °C – Dissociation half‐life	20 ± 3 days	4 ± 0.5 days	18 h	4 h	NA	<5 s	<5 s

Beyond the very crucial need for high stability, other main features are required in the design of an optimal GBCA to be employed in clinical practice, like i) high aqueous solubility, ii) fast biodistribution and excretion, iii) good contrast efficiency at small dosages, and iv) a good biological safety profile.

Once intravascularly injected, commercial GBCAs quickly diffuse in the vascular compartment and extravasate in the extravascular/extracellular compartment of districts with a leaky endothelium (e.g., in the tumor).^[^ ^31^ ^]^

In the absence of acute kidney injury, they are excreted by the renal route with *t*_1/2_ of the order of 1–2 h. Overall, the use in vivo was considered very safe.^[^ ^32^ ^]^

**Figure 3:**
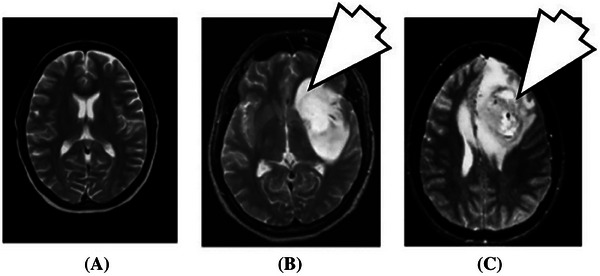
Brain MRI acquisition reporting A) normal brain, B) benign tumor, and C) malignant tumor. Adapted from Tandel et al. (2019).

In the regions in which they distribute, they act by shortening the *T*_1_ of bulk water protons, brightening the MR image of that region, and highlighting the diseased tissue. They are typically employed to visualize tumor burdens and obtain insights into tumor perfusion and vessels’ permeability. Providing an example, GBCAs can help distinguish malignant from benign brain tumors, since they can provide insights into the degree of tumor perfusion (**Figure** **3**).^[^ ^33^ ^]^

## 2 Drawbacks Related to the Use of GBCAs

### 2.1 Gadolinium Retention and Toxicity in Humans

Albeit GBCAs were considered totally safe for humans at first, different evidence arose over the years about their accumulation and pathogenic role. In the early 2000s, the only adverse effect ascribed to Gd(III) use was related to the acute risks of allergic reactions, with no significant differences with respect to the iodinated contrast agents, commonly used for X‐ray computed tomography.^[^ ^34^ ^]^ Since the beginning of 2006, there has been evidence of Gd(III) involvement in nephrogenic fibrotic dermopathy (NFD) and nephrogenic systemic fibrosis (NSF) pathogenesis.^[^ ^35^ ^]^ NFD is a rare, acquired, and idiopathic skin disease observed in patients with renal disorders, it leads to thickness and induration of the skin, sometimes with nodule formation. NFD diagnosis occurs after biopsy when typical histopathologic features are found, like fibroblast proliferation and elastic fibers, mucin deposition, and collagen remodeling.^[^ ^36^ ^]^

**Figure 4:**
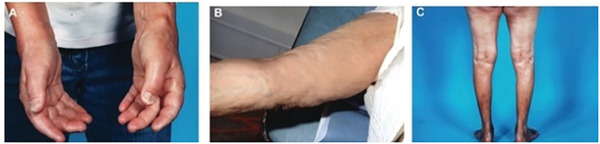
Clinical photos from patients with nephrogenic systemic fibrosis. A) Tightness and hardness of the hands combined with joint contractures. B) Firm nodules establishing a cobblestone configuration. C) Tight and firm skin on lower legs. Adapted from Elmholdt et al. (2013).

NFD is sometimes accompanied by systemic fibrosis (NSF), meaning that the fibrotic phenomenon manifests in internal organs like the lungs, liver, muscles, and heart, with very severe effects on human health (**Figure** **4**).^[^ ^31^ ^]^

In 2006 Grobner^[^ ^35^ ^]^ concluded that patients with acute renal failures undergoing MRI with GBCAs (in particular Gd(III)‐DTPA, Magnevist) in specific conditions could develop NSF disease.

**Figure 5:**
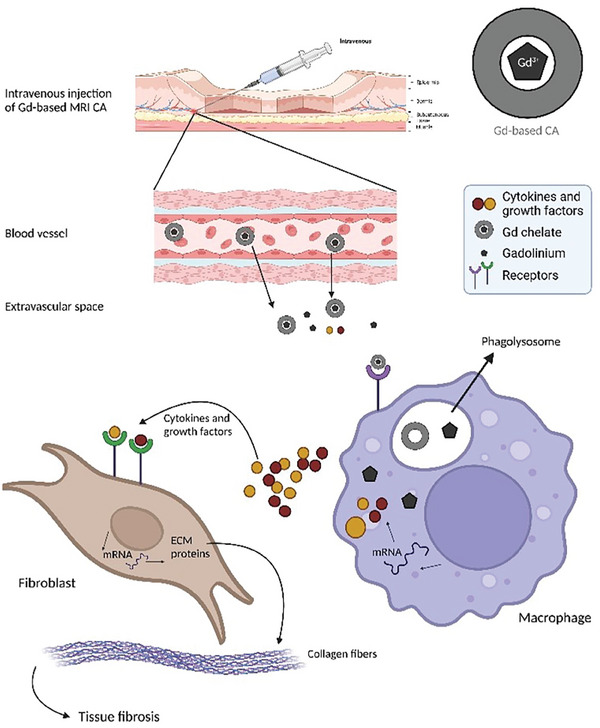
Representation of the profibrotic mechanism of gadolinium. The chelated gadolinium in MRI‐CA is injected intravenously. The escape in the extravascular space happens due to high concentration and increased retention owing to renal insufficiency. Macrophages exposed to the extravascular Gd are induced to produce and secrete proinflammatory/profibrotic cytokines. This induces the activation of dermal fibroblasts, resulting in a higher expression of genes encoding interstitial collagens ECM molecules. This culminates in tissue fibrosis in subjects with renal insufficiency.

The most subscribed hypothesis about Gd(III)’s role in NFS etiopathogenesis was the dechelation/transmetallation theory: in patients with renal failure disorder, the consequent GBCAs increased half‐life could induce the release of the free ion form from the chelators causing a toxic effect.^[^ ^37^ ^]^ Moreover, it was demonstrated in vitro how, upon the endosomal uptake of Gd(III) complexes by fibroblast and macrophages, the acidic lysosomal environment promoted the degradation of some linear complexes (Gd(III)‐DTPA‐BMA or Gd(III)‐DTPA^2‐^), with the deposition of Gd(III) ions as phosphates after some hours from the entrapment, while macrocyclic complexes, like Gd(III)‐HPDO3A, were unaffected and thus more stable (**Figure** **5**).^[^ ^38^ ^]^

A possible mechanism to tentatively explain this degradation implies the structural modification of linear complexes due to N‐oxidation of the acetic‐coordinating arm.^[^ ^39^ ^]^ This effect can be strongly enhanced inside macrophages endolysosomes and phagosomes where acidic pH and oxidative environment, as well as a harsh enzymatic armory, are present able to attack and degrade the xenobiotic molecules.

Therefore, in people with acute kidney injuries, the association of increased GBCAs half‐life and the state of inflammation, with macrophage activation, can increase the Gd(III) release from the complex and the subsequent toxic effects associated with it.^[^ ^30^, ^31^ ^]^

Besides the NSF, there is emerging evidence about Gd(III) retained in the central nervous system and other organs of patients repeatedly exposed to GBCA administrations. Indeed, in those patients, an increased signal intensity in noncontrasted *T*_1‐weighted_ MRI scans was highlighted.^[^ ^40^ ^]^ This hyper intensity was associated with the retention of tiny amounts of Gd(III) and deposition in the brain and other tissues even in the presence of normal renal function.^[^ ^34^ ^]^ Even though such findings were not associated with histopathological changes and clinical consequences, they raise some concerns about Gd(III) safety, the stability of the chemical form (macrocycle or linear), and their permeability through the blood–brain barrier and deposition in tissues.

### 2.2 European Medicines Agency (EMA) and FDA Safety Evaluations

The issue of NSF onset and, above all, Gd(III) deposition and retention prompted the EMA and FDA to send alerts and require caution for the clinical use of GBCAs.^[^ ^37^ ^]^

Even if there is no evidence that Gd(III) deposition in the brain has ever caused any harm to patients, EMA has recommended restrictions and suspension for some linear agents to prevent any potential risks.

On the contrary, no problem is related to the use of macrocyclic agents (gadobutrol, gadoteric acid, and gadoteridol) since they have a high stability, and the release of Gd(III) is strongly lower than linear agents. However, the recommendation is to use the lowest dose able to provide enough contrast in the image and only if noncontrasted MRI is not sufficient for the clinical request.

Over the years, the U.S. FDA has published several Drug Safety Communication regarding GBCAs.

So, there was no evidence of potential health harm due to Gd(III) accumulation in patients associated with GBCAs administration, even with the compounds that showed higher retention in the body. The indications for the clinical use were unchanged, highlighting the importance of a rigorous evaluation of the specific medical needs before the prescription of a contrasted MRI scan.

### 2.3 GBCAs as Water Pollutants: First Evidence, Routes, and Drawbacks

Even if EMA and FDA have required caution on GBCAs use, they strongly remain needed in clinical practice. The increase in MRI scans using GBCAs for diagnosis has significantly raised the total amount of Gd(III) used. Between 1998 and 2008, global Gd(III) use increased nearly tenfold (from 20 million applications in 1998 to 150–180 million in 2008), with ≈5% (180–220 tons per year) used for diagnostics.

The global MRI systems market reached $4.61 billion in 2021, with an expected growth of 6.9% during the forecast period, driven by technological advancements and increased MRI adoption. This rise is linked to the higher number of Gd(III)‐contrasted MRI exams, the primary source of anthropogenic Gd(III) in the environment. From the first GBCA approval (Gd(III)‐DTPA) in 1988 to September 2009, about 100 million applications were performed worldwide, with 22–66 tons of Gd(III) used annually for contrast agents (20 million applications per year, each using 1.1–3.3 g of Gd(III)).

An abnormally high concentration of Gd(III) in surface waters, groundwater, and tap water was reported for the first time in 1996 by Bau and Dulski,^[^ ^41^ ^]^ who conducted a crucial study about REEs diffusion in the hydrosphere of the Berlin area. Further research brought to light positive anthropogenic Gd(III) anomalies as a more diffused phenomenon, either in Europe, Asia, North America, and Australia. Kulaksiz and Bau analyzed rivers in the above‐spoken areas reporting cases of both normal and abnormally increased concentrations of Gd(III).^[^ ^42^, ^43^ ^]^

**Figure 6:**
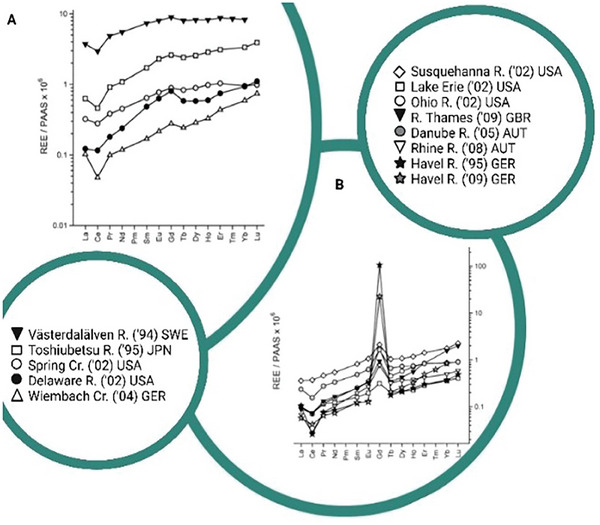
REE distribution profile in water samples collected from rivers (R.) from different countries and creeks (Cr.). Comparison among A) regions anthropogenic Gd free and B) urbanized area with high antropogenic Gd presence. Data from Bau and Dulski^[^ ^41^ ^]^ and Kulaksız and Bau.^[^ ^42^ ^]^

**Figure** **6** shows Post Archean Australian Shale (PAAS)^[^ ^44^ ^]^ data normalization, reporting a very high increase of Gd(III) in Havel River (Berlin Area) that increased about five times in 14 years (from ≈20 × 10^6^ Gd(III)/PAAS in 1995 to ≈100 × 10^6^ Gd(III)/PAAS in 2009). Once administered, GBCAs are removed through urine within 24–48 h in patients with functional kidneys.^[^ ^45^ ^]^ Accordingly, they have been detected in hospital effluents and urban wastewater treatment plants effluents, through which they reach surface waters, leading to a large increase in concentration of Gd(III). Interestingly, the concentration of Gd(III) does not decrease significantly during wastewater treatment, both due to the chelates’ high solubility and resistance to microbial degradation and ineffective wastewater filtering and decontamination systems.^[^ ^45^ ^]^ Thus, Gd(III) complexes easily reach rivers and lakes and could migrate to the hydrosphere. It is also important to consider that geogenic Gd(III), like other REEs, is highly particle‐reactive, and could aggregate precipitating with, due to salting out at the estuary. On the contrary, anthropogenic Gd(III) complexes are characterized by high stability and nonreactivity thus, such abnormal GBCAs amounts are easily transferred to coastal waters. A positive Gd(III) abnormal concentration has been detected in bays of the North Sea,^[^ ^43^ ^]^ the Mediterranean Sea,^[^ ^46^ ^]^ Pacific Ocean at San Francisco (USA),^[^ ^47^ ^]^ Nagoya (Japan),^[^ ^48^ ^]^ and Brisbane (Australia).^[^ ^49^ ^]^

Another important consideration to be carried out is that Gd(III) complexes cannot be removed by the environment by aquatic plants. In fact, it is well known that aquatic plants have a generally high potential in the elimination of pollutants in an effective and quite inexpensive fashion, and in the accumulation (and decontamination) of heavy metals (e.g., Cr, Cd, Ni, U, Pb, etc.). For this reason, several studies were focused on investigating whether phytoremediation could be a solution for the removal of Gd(III) chelates from wastewater.^[^ ^45^, ^50^, ^51^, ^52^ ^]^ However, all the most used macrophyte species showed not to impact the Gd(III) concentration of water, with no relevant accumulation of Gd(III) complexes in the plant bodies.

Caution on the use of GBCAs has to be carried out starting from all the above‐reported considerations, i.e., i) the increased number of executed clinical Gd(III)‐contrasted MRI scans (corresponding to an enhanced use of Gd(III)), ii) the elimination of Gd(III) complexes through the patients’ urines in the city sewage systems, iii) the high stability and inertness of Gd(III) complexes with consequent long environmental half‐life, iv) the difficulty of blocking Gd(III) complexes by aquatic plant but also by artificial filters and decontamination systems. Hence, the problem of environmental accumulation of Gd(III) species and their effect on flora and fauna (especially of water ecosystems) is not neglecting.^[^ ^53^, ^54^ ^]^

## 3 Gd(III) Accumulation in Water Flora and Fauna

**Figure 7:**
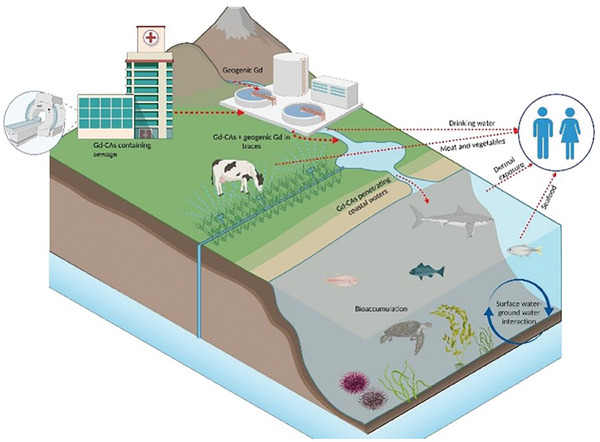
Gadolinium spreading through the water cycle, bioaccumulation in animals and plants and how the contamination reaches the humans.

Considering Gd(III) ion toxicity and its high concentration in contaminated water, the potential bioaccumulation of Gd(III) chelates in organisms and plants represents a risk for either the development or survival of the biota, beyond the possible human exposure through the food and drink chain (**Figure** **7**).

### 3.1 Effects on Flora

The first major point regarding the ecological toxicity of Gd(III) ions in plants is its bioaccumulation through contaminated water and soil, with controversial results and findings. With the aim of testing potential biofilters for the elimination of different Gd(III) chelates (GBCAs) from water, Braun et al. in 2018^[^ ^45^ ^]^ investigated the role of four species of aquatic macrophytes (*Lemna gibba*, *Ceratophyllum demersum*, *Elodea nuttallii*, and *Elodea canadensis*) notoriously known to accumulate heavy metals and commonly used for removing pollutants.

Surprisingly, there was no significant accumulation of Gd(III) in these aquatic plants, thus also highlighting a reduced risk in the accumulation of anthropogenic Gd(III) in the food chain.^[^ ^45^, ^52^ ^]^

Lindner et al.^[^ ^55^ ^]^ focused their research on the potential uptake of GBCAs (Dotarem, Gadovist, and Multihance) by the root system of *Lepidum sativum* (cress plants).

The considerable amounts of Dotarem and Gadovist uptaken by them, lead to the hypothesis of an uptake through the apoplastic pathway, due to the incomplete establishment of the Casparian band (a structure that prevents the uptake of large molecules functioning as a filter) in the young tips of the roots.^[^ ^56^, ^57^ ^]^ Furthermore, speciation analyses highlighted how cress plants were able to take up and transport to their leaves the contrast agents (but not their metabolites) with no or few unimportant modifications. These latter findings seem to be confirmed by further analysis conducted by Lingott et al.^[^ ^58^ ^]^ that, through the use of laser ablation inductively coupled plasma mass spectrometry, were able to demonstrate that plants such as *Lemna minor* (duckweed), *Lepidium sativum* (cress plant), and *Zygnema* (filamentous algae), if cultivated in water or soil contaminated by Gd(III)‐CAs, will uptake Gd(III)‐CAs such as Omniscan and Magnevist.^[^ ^58^ ^]^

Furthermore, confirmation of the risk of Gd(III) involvement in the food chain by assessing bioaccumulation of REEs, Gd(III) included, in wheat (*Triticum aestivum*), highlighted a major presence of those elements in the roots rather than in steam and leaves.^[^ ^59^ ^]^ Not only the food supply chain may be involved, but the ecological effect of REEs (especially Gd(III)) in plants is majorly represented by the inhibition of the uptake of essential nutrients for plants, like Ca(II) due to their similarity in the ionic radii and charge density. Indeed, even in plants, lanthanides can interfere with different functions in which Ca(II) ions play an essential role, from cell wall formation to flowering or photosynthesis.^[^ ^60^ ^]^ Moreover, risks regarding the interference of Gd(III) in the development and metabolism of plants have still to be further described.

### 3.2 Effects on Fauna

Contaminated water plants represent a dangerous element for Gd(III) passage from water to organisms and furthermore potentially to humans. Revel et al. highlight that REEs, commonly used in high‐tech applications, accumulate in water systems and can influence aquatic organisms by altering enzymatic activities, oxidative stress responses, and reproduction.^[^ ^61^ ^]^ Lingott et al. showed how *Daphnia magna* (water flea), extremely attractive for fish, was able to accumulate gadopentetic acid (Magnevist) if exposed to contaminated water or nourished with *Scenedesmus subspicatus* (specific one‐celled algae) cultivated in a Gd(III)‐containing nutrition medium.^[^ ^58^ ^]^

Moreover, bioaccumulation in the skin and intestine was detected in *D. magna* exposed to a contaminated medium. The uptake via nutrition caused strong Gd(III) accumulation in the intestine, highlighting the risk of Gd(III)‐CA contamination in the food chain of higher organisms. Gd(III) toxicity acts also on the development of specific organisms. Martino et al.^[^ ^62^ ^]^ investigated sensitivity across four species of sea urchins: *Paracentrotus lividus* and *Arbacia lixula* from Europe and *Heliocidaris tuberculata* and *Centrostephanus rodgersii* from Australia. Considering the Gd(III) ion potential action as a blocker of Ca(II) channels, due to similarities in ionic radius,^[^ ^21^ ^]^ the exposition to Gd(III) acetate tetrahydrate (GAT), affected the development of both embryos and skeleton growth in sea urchins with a variable sensitivity across the four species that showed an EC_50_ (half maximal effective concentration, where 50% of Gd(III) maximal effect is observed) between 56 and 132 nm.^[^ ^62^ ^]^

**Figure 8:**
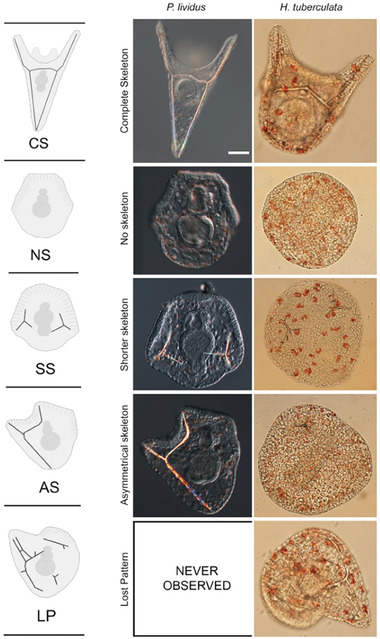
Effect of Gd(III) exposure on the development of Paracentrotus lividus (middle column) or Heliocidaris tuberculate (right column). On the left five morphotypes observed and categorized based on skeleton occurrence, abnormality, and asymmetry (CS, complete skeleton; NS, no skeleton; SS, shorter skeleton; AS, asymmetrical skeleton; LP, lost pattern). In the first row there are sample w/o Gd(III) exposure. In the rows 2–5 there are sample after exposure to Gd(III). Adapted from ref. [62].

The effects of Gd(III) on *Paracentrotus lividus* (sea urchin) embryos were focused on different pathways of sea urchin development: morphogenesis, biomineralization, and stress response through autophagy (**Figure** **8**).^[^ ^62^ ^]^

Authors observed a delay of biomineral deposition at 24 h postfertilization, and a strong impairment of skeleton growth at 48 h postfertilization, frequently displayed by an asymmetrical pattern. Moreover, the mesodermal cells designated for biomineralization were found not correctly migrated at 48 h postfertilization. Finally, an increased number of autophagolysosomes and autophagosomes was found.^[^ ^62^ ^]^

Recently, Pagano et al. reported a significantly decreased fertilization rate in sperm of sea urchin exposed to very low concentration of some earth elements like cerium (Ce) and lanthanum (La) or their combination.^[^ ^63^ ^]^

## 4 How to Solve this Issue? The 4R's of Gd(III)

**Figure 9:**
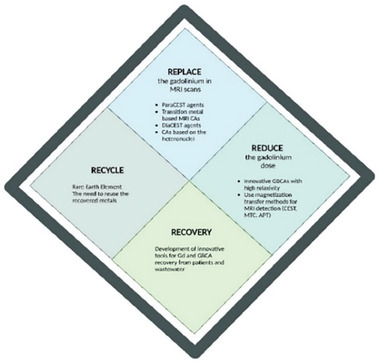
The 4R's of Gd(III), a summary.

Also, the accumulation of Gd(III) in the environment as a contaminant is important, with several effects, especially on aquatic fauna. Moreover, anthropogenic Gd(III) was found in tap water and in tap water‐based beverages from fast‐food franchises in six major cities in Germany.^[^ ^64^ ^]^ Hence, a 4′R proposal (**Figure** **9**) can be considered, i.e., i) Replacing GBCA with other MRI CAs, ii) Reducing the dose of administered GBCAs in MRI scans and iii) Recovering Gd(III) from the environment and Recycling.

### 4.1 Replacing the Gd(III) in MRI Scans

The first proposal to overcome the drawback related to the use of GBCAs (both in human health and in the environment) is replacing Gd(III)‐based MRI CAs with other MRI CAs:, e.g., transition metal complexes (mainly based on Fe(III) or Mn(II)) or developing free‐metal CAs (e.g., diamagnetic chemical exchange saturation transfer (CEST) agents. An incredibly huge number of studies have been done with the aim of exploiting new MRI contrast agents without Gd. Four main classes of Gd(III)‐free MRI CAs can be considered.

1. ParaCEST agents based on other lanthanides’ ions.
2. Transition metal (Fe(III), Mn(II))‐based MRI CAs generating contrast based on CEST or relaxation (*T*_1_/*T*_2_) mechanism.
3. Diamagnetic chemical exchange saturation transfer (DiaCEST) agents that do not employ metals (e.g., sugars, amino acids, etc.).
4. CAs based on the heteronuclei (^13^C, ^15^N, ^19^F, ^23^Na, ^129^Xe, …), eventually using hyperpolarization.

Following a brief overview of the above reported four classes of Gd(III)‐free MRI CAs is reported to highlight their pros and cons.

#### 4.1.1 ParaCEST Agents Based on Other Lanthanides’ Ions

ParaCEST agents based on lanthanides are a widely exploited class of MRI CAs.^[^ ^65^, ^66^, ^67^, ^68^, ^69^, ^70^, ^71^, ^72^, ^73^ ^]^ Very good results have been reached at the preclinical level and some of these chemicals are under examination for translation in human studies.^[^ ^10^, ^21^, ^66^, ^67^, ^68^, ^74^ ^]^

The contrast generation relies on the chemical exchange of protons between the lanthanide complex and surrounding water molecules, resulting in a detectable change in signal intensity. This approach offers several advantages over traditional CAs, including:

- On–off contrast: the contrast is switched‐on only upon presaturation at specific resonance.
- Multiplexing capabilities: since different CEST agents resonate at distinct frequencies, it is possible to image multiple targets simultaneously (multicolor MRI).
- Chemical specificity: they can be designed to detect specific functional groups or molecules, making them excellent for studying biochemical processes.
- Dynamic contrast (responsive agents): they can be responsiveness to environmental parameters allows monitoring of pH, temperature, enzyme activity, or specific biomolecules in real‐time, providing functional information beyond anatomical details.

Lanthanides, such as europium (Eu(III)), dysprosium (Dy(III)), and ytterbium (Yb(III)), possess favorable electronic and magnetic properties that make them well‐suited for paraCEST imaging.^[^ ^75^, ^76^, ^77^, ^78^ ^]^ ParaCEST agents also offer multiplexing capabilities, enabling the simultaneous detection of multiple targets or molecular species (multicolor MRI).^[^ ^79^, ^80^, ^81^ ^]^ By designing lanthanide complexes with distinct exchangeable protons, different agents can be employed and distinguished based on their specific CEST frequencies. Moreover, paraCEST agents can be engineered to target specific biomolecules or cellular processes, further expanding their applications. They can be tailored to respond to parameters of pathological interest such as pH or enzyme activity, enhancing the specificity and accuracy of diagnostic imaging. A notable area of research focuses on pH‐responsive paraCEST MRI contrast agents.^[^ ^66^, ^71^, ^72^, ^82^ ^]^

A very interesting field of study relies on the design of pH‐responsive paraCEST MRI CAs. Just to cite an example, YbHPDO3A has been designed and tested as CA able to map pH in the extravascular/extracellular region of the tumor.^[^ ^71^, ^83^ ^]^ This CA appeared to be very interesting in view of a possible clinical translation since it is the chemical analogue of the clinically approved gadoteridol (ProHance, Bracco Imaging S.p.A.) where Gd(III) is replaced with Yb(III). Hence, the same biocompatibility and safety profile is reported, with a high potential for clinical translation.

However, even if they can add significant information about the analyzed diseases, their application is hampered by the presence of the same drawbacks reported for Gd(III). Their deposition/accumulation in organs and environmental contamination is presumed to be like the one reported for Gd(III) but potentially more extensive due to the higher dosage required for paraCEST detection compared to traditional GBCAs. The sensitivity of CEST MR imaging is, in the millimolar order, whereas the sensitivity of *T*_1_ relaxation‐based MRI is in the tens/hundreds micromolar order. Serious caution must be devoted toward their development and in vivo application.^[^ ^84^ ^]^

As above reported, the response of CEST agents is very sensitive to the environment (pH, ionic strengths, etc.). This issue is very powerful in obtaining responsive agents, but it requires caution in setting proper CEST experiments, especially in vivo where these different parameters cannot be easily controlled and maintained constant. Moreover, in vivo, the large magnetization transfer effect of tissues can strongly influence the detection of CEST agents, when they are not highly shifted (e.g., Eu complexes).

#### 4.1.2 Transition Metal (Fe, Mn(II))‐based MRI CAs

Transition metal MRI CAs, such as iron (Fe) and manganese(II) (Mn) complexes offer an alternative to GBCAs due to their paramagnetic properties and greater biocompatibility.^[^ ^10^, ^85^, ^86^, ^87^, ^88^, ^89^ ^]^ Fe and Mn(II) are largely present in the human body and in the earth's crust. Fe and Mn(II)‐based contrast agents have garnered significant interest due to their distinct properties and potential applications in MRI.^[^ ^85^, ^87^, ^90^, ^91^, ^92^ ^]^ Mn(II)‐based contrast agents, like manganese chloride (MnCl_2_) and manganese dipyridoxyl diphosphate, generate positive contrast in *T*_1‐weighted_ images and are valuable for functional brain MR imaging, myocardial perfusion studies, and hepatobiliary imaging. Mn(II) ions can cross cell membranes via voltage‐gated calcium channels, making them ideal for neuronal tracing and mapping. Once inside neurons, they distribute throughout the cell bodies, dendrites, and axons, reflecting neuronal activity and connectivity. Mn(II)‐enhanced MRI (MEMRI) is widely used in neuroscience research to map brain function and circuitry by tracking Mn(II) ions along neural pathways.^[^ ^95^, ^96^, ^97^, ^98^ ^]^

A nice example of MEMRI application was proposed by Van der Linden et al., who extensively applied this methodology to image brain plasticity in songbirds.^[^ ^93^, ^94^ ^]^

More recently, another very interesting application of MEMRI in the study of brain development was reported by Bifone and co‐workers. They applied MEMRI to study the effect of sensory stimulation to the embryo (in utero or in ovo) of domestic chicks, showing that light‐induced brain asymmetry can arise when proper light stimulation is applied.^[^ ^95^ ^]^

MEMRI has also been employed in studies investigating neurological disorders, such as epilepsy, Alzheimer's disease, and Parkinson's disease.

In addition to neuroscience research, MEMRI has found applications in other fields. For instance, in cardiology, Mn(II)‐based CAs have been used to evaluate myocardial viability and assess cardiac functions.

While MEMRI provides valuable insights, there are certain considerations and limitations to be aware of. Mn(II) CAs, although offering unique advantages, have challenges related to their short plasma half‐life, rapid systemic clearance, and dose‐dependent neurotoxicity. Furthermore, Mn(II) ions can compete with endogenous Ca(II) ions, affecting calcium signaling and potentially altering neuronal function.

The other transition metal used in MRI CA is Iron.^[^ ^13^, ^86^, ^96^, ^97^, ^98^ ^]^ Fe(III)‐based contrast agents, such as superparamagnetic iron oxide nanoparticles (SPIO NPs), possess high relaxivity and excellent capabilities to generate negative contrast (darkening of the image) in *T*_2‐weighted_ MR imaging.^[^ ^99^ ^]^ They have been extensively used for vascular imaging, tumor detection, and tracking cellular processes.

Due to their small size, typically ranging from 10 to 100 nm, they exhibit superparamagnetism, meaning that in the absence of an external magnetic field, their magnetic moments are randomly oriented.^[^ ^100^ ^]^

One of the main applications of SPIO CAs is in the detection and characterization of liver lesions, particularly in cases of liver cancer and metastases. They are taken up by liver cells, leading to a decrease in signal intensity on *T*_2_*_‐weighted_ MR images.

Additionally, SPIO nanoparticles have been investigated for targeted imaging and drug delivery applications.^[^ ^101^ ^]^ By attaching specific ligands or antibodies to the nanoparticle surface, SPIO agents can be directed toward specific molecular targets, such as tumor markers.

Despite their advantages, there are certain limitations to consider when using SPIO CAs. The small size of the particles can result in rapid clearance from the bloodstream, limiting the imaging time window.

In recent years, the development of newer contrast agents, such as ultrasmall SPIO (USPIO) nanoparticles and Ferumoxytol (Feraheme) originally approved by the FDA in 2009 for IV treatment of iron deficiency in adults with chronic kidney disease,^[^ ^102^ ^]^ has addressed some of these limitations. USPIO nanoparticles exhibit longer circulation times, allowing for extended imaging periods, while Ferumoxytol, a USPIO agent approved for clinical use, has shown promise for various applications, including vascular imaging and inflammation detection.^[^ ^103^, ^104^ ^]^

While Fe(III) contrast agents have been extensively studied and approved for clinical use, there are concerns regarding their long‐term safety, potential for Iron overload, and limited relaxivity at high magnetic field strengths.

Alternatively to (U)SPIO NPs, also iron‐based complexes, such as Fe‐EDTA and other low‐molecular‐weight high spin Fe(III) coordination compounds, are gaining attention as safer effective alternatives to GBCAs for MRI.^[^ ^105^, ^106^, ^107^, ^108^ ^]^ Mononuclear and multinuclear Fe(III) complexes have demonstrated high relaxivity and stability and exhibit a favorable safety profile while maintaining comparable imaging efficacy to GBCAs.

Fe‐EDTA and derivatives modified with hydrophilic ligands have shown promise due to their solubility and reduced risk of generating reactive oxygen species under physiological conditions. Moreover, Fe(III) complexes benefit from adjustable redox properties, which are critical for minimizing side effects such as oxidative stress. The design of these compounds often incorporates ligands like catechol and carboxylates to optimize relaxivity and stability while preventing hydrolysis or reduction in vivo. These attributes position Fe complexes as potential candidates for broader clinical adoption, particularly for patients with contraindications to Gd‐based agents.^[^ ^109^ ^]^

#### 4.1.3 diaCEST Agents

For sure, CAs w/o metals can be seriously considered as a valid alternative to GBCAs, eliminating the drawbacks related to metals, whether we are talking about transition metals or lanthanides. Therefore, the third class relies on diaCEST MRI contrast agents.^[^ ^74^, ^110^, ^111^, ^112^, ^113^, ^114^, ^115^, ^116^, ^117^ ^]^ Unlike traditional paramagnetic CAs, diaCEST agents do not contain unpaired electrons and do not induce significant changes in the local magnetic field. Instead, they exploit the exchange of protons between the agent and the surrounding water molecules, resulting in the generation of MRI contrast. Preclinical research on diaCEST CAs is ongoing and very nice examples of applications have been reported for theranostic studies on murine models of diseases, for the detection of enzyme activity, for metabolic imaging, for mapping pH, for imaging of glycosylation, etc.^[^ ^74^, ^114^, ^115^ ^]^

One of the most important applications of diaCEST is for metabolic imaging of tumors. Glucose CEST agents have been developed to monitor glucose metabolism in tumors, providing valuable information about tumor aggressiveness and response to therapy.^[^ ^118^, ^119^, ^120^ ^]^ Additionally, other metabolites such as lactate, glutamate, and glutamine can be targeted using CEST agents to study metabolic alterations associated with diseases like cancer and neurodegenerative disorders.^[^ ^121^, ^122^, ^123^ ^]^

Another wide exploited application of diaCEST relies on pH imaging. pH‐responsive diaCEST agents exploit pH‐dependent chemical exchange properties, allowing for the assessment of pH variations within biological tissues. pH imaging with diamagnetic CEST agents has shown potential in characterizing tumor microenvironments, where acidic pH is associated with tumor aggressiveness and treatment resistance.^[^ ^13^, ^71^, ^123^ ^]^

The most important drawback, that hampers their application in comparison to GBCAs, is related to the lower sensitivity, because a tens millimolar concentration of diaCEST agents is required to reach the detection threshold, i.e., 1–2 orders of magnitude higher than the one of Gd(III) complexes.

#### 4.1.4 CAs‐Based on the Heteronuclei

Heteronuclear MRI, which uses nuclei other than protons (^1^H), is a promising class of Gd(III)‐free MRI contrast agents. Nuclei such as carbon‐13 (^13^C), nitrogen‐15 (^15^N), fluorine‐19 (^19^F),^[^ ^124^ ^]^ sodium‐23 (^23^Na), xenon‐19 (^129^Xe), offer unique insights into biological tissues and processes due to their specific properties.^[^ ^125^, ^126^, ^127^ ^]^ Heteronuclear MRI allows for probing different aspects of tissue composition, metabolism, and function. For instance, ^13^C MRI can track metabolic processes in vivo by following the conversion of hyperpolarized ^13^C‐labeled substrates, providing valuable insights into cellular metabolism with potential applications in studying cancer, cardiac function, and brain metabolism.^[^ ^128^ ^]^

Similarly, ^15^N and ^19^F MRI offer opportunities to study specific molecules or compounds, such as imaging protein dynamics or monitoring the distribution of fluorinated contrast agents for targeted imaging.^[^ ^129^ ^]^

^23^Na MRI is a specialized imaging technique that focuses on the visualization and quantification of sodium within biological tissues. Although sodium is present in lower concentrations compared to hydrogen protons (^1^H), it offers unique insights into cellular viability, tissue integrity, and various physiological processes.

One of the primary applications of ^23^Na MRI is in the assessment of tissue viability, particularly in ischemic events, stroke, and cardiovascular diseases.

Furthermore, ^23^Na MRI has been employed to investigate various pathological conditions, including tumors and edema. Changes in sodium ion concentrations within these tissues can indicate alterations in cellularity, extracellular space, and ion transport mechanisms. Moreover, sodium ions play a crucial role in neuronal signaling, and ^23^Na MRI has been applied to study brain function and neurological disorders. By visualizing sodium concentrations in the brain, researchers can gain insights into cellular metabolism, ion homeostasis, and neuronal activity. This approach has shown promise in conditions such as epilepsy, multiple sclerosis, and brain tumors.^[^ ^130^, ^131^ ^]^

Despite its advantages, ^23^Na MRI presents certain challenges. Sodium has a relatively short *T*_2_ relaxation time, leading to reduced signal intensity and limitations in spatial resolution.

Another interesting heteronuclear for MRI is represented by ^129^Xe.^[^ ^126^, ^127^ ^] 129^Xe MRI is an intriguing and rapidly evolving technique that utilizes hyperpolarized xenon gas as a CA for imaging, because of the high solubility and chemical shift sensitivity of xenon gas. It is used to study lung function, gas exchange, and blood flow, offering great potential in several areas of research and clinical applications. By imaging the distribution and movement of hyperpolarized ^129^Xe gas within the lungs, it is possible to assess lung ventilation and gas diffusion, providing valuable insights into conditions such as chronic obstructive pulmonary disease, asthma, and pulmonary fibrosis.

Additionally, ^129^Xe MRI can be used to study other organs and tissues beyond the lungs. For example, investigations have been conducted on brain imaging, where xenon can act as a blood flow tracer and provide information about cerebral perfusion.

However, there are some limitations and challenges associated with ^129^Xe MRI.^[^ ^132^ ^]^ One major hurdle is the requirement for hyperpolarization techniques to enhance the signal of the xenon gas, as its natural abundance is low and the polarization decays rapidly.

Despite their advantages, heteronuclear MRI techniques also present challenges and important limitations. One major drawback is the lower natural abundance and lower sensitivity of these nuclei compared to protons. This necessitates the use of specialized hardware and imaging sequences to enhance the signal‐to‐noise ratio.

### 4.2 Reducing the Gd(III) Dose

An alternative to replacing Gd in MRI contrast agents is developing new Gd(III)‐based agents with higher relaxivity, allowing for lower doses and benefiting patients and the environment.

Innovative GBCAs have been designed to maximize the interaction between Gd(III) ions and water molecules, enhancing contrast.^[^ ^8^, ^10^, ^21^, ^100^, ^133^ ^]^ Various strategies have been employed to achieve high relaxivity, including the design of macromolecular structures or the use of NPs which allow the accumulation of a larger number of Gd(III) ions in the site of interest, making possible the detection of the disease.

Macromolecular Gd(III)‐based agents link multiple Gd(III) ions to polymers or dendrimers, resulting in higher relaxivity and better contrast enhancement. They also prolong circulation time and retention in target tissues. Nanoparticle‐based Gd(III) agents use various materials to carry Gd(III) ions, achieving high local concentrations and increased relaxivity, with added functionalities like targeting ligands or drug delivery.

Optimizing the properties of Gd(III) complexes for better contrast involves understanding parameters such as water coordination number (*q*), water residence time (*τ*_M_), and rotational correlation time (*τ*_R_).

Following a simple brief description of these parameters. The water coordination number (*q*) refers to water molecules directly coordinated to Gd(III) ions. The protons of these molecules relax more efficiently than those of water molecules, not coordinated. Thus, enhancing the *q* number is important in improving relaxivity. An example is provided by the commercial gadopiclenol, which displays a *q* = 2, higher than other conventional GBCAs, which generally display *q* = 1 (e.g., ProHance, Dotarem, etc.).^[^ ^27^ ^]^

The water residence time (*τ*_M_) is another important parameter in determining the efficiency of a contrast agent for MRI. Hence the optimization of water exchange rate (*k*_ex_ = 1/*τ*_M_) is necessary to have good GBCAs. *τ*_M_ values for good GBCAs are in the 1–100 ns range. However, this parameter is less important since a broad range of water exchange rates have been reported to be suitable for reaching good values of relaxivity, at least for small and medium size molecules.

The rotational correlation time (*τ*_R_) reflects the rotational motion of the contrast agent. Most of the clinically approved GBCAs are based on DTPA or DOTA ligands and display molecular weights of 550–900 Da, providing *τ*_R_ values of ≈60–120 ps at room temperature. Slowing down the rotational motion by increasing the molecular weight or size of the agent can enhance relaxivity, leading to more efficient relaxation processes in the 20–60 MHz range, typically used in clinical MRI scanners. As above reported, several determinants significantly impact the relaxivity of Gd(III)‐based MRI contrast agents and optimizing them through molecular design, ligand selection, and structural modifications can improve contrastographic properties and enhance relaxivity. By carefully manipulating these determinants, researchers can develop GBCAs with improved relaxivity, leading to enhanced image contrast, better visualization of anatomical structures, and more accurate diagnoses in the field of medical imaging but, above all, allowing reducing the dose of needed Gd(III) with positive outcomes for patients and environments.

Normally, GBCAs are detected by MRI simply exploiting its capability to shorten *T*_1_ of tissue water. However, other effects of Gd(III) on water proton MRI signal can be considered. In 2015 our group reported “the possibility of using magnetization transfer contrast for an improved MRI detection of *T*_1_ relaxation agents”.^[^ ^134^ ^]^ The method is based on the *T*_1_ dependence of magnetization transfer (MT) contrast. The reported data showed that in cellular experiments the MTC method displays a better sensitivity with respect to the common *T*_1W_ experiments.

**Figure 10:**
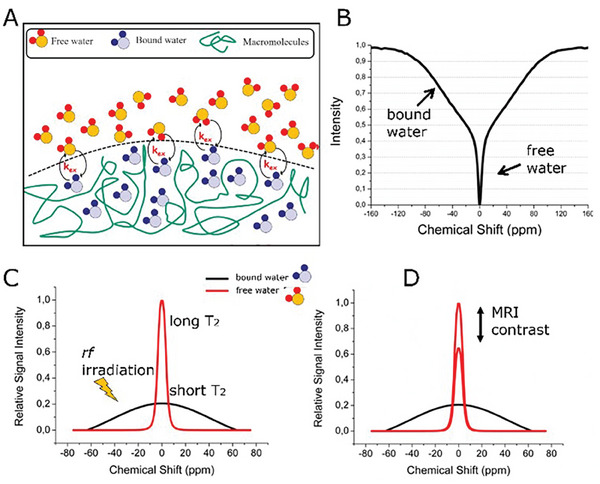
Scheme of the MTC process. A) Exchange between the free bulk water pools and bound water molecules. B) A representative Z‐spectrum of water MRI signal. C) Relative signal intensity of the two pools reported in (A). D) Difference in relative signal intensity generating the observable MRI contrast.

MTC in MRI was described by Wolff and Balaban^[^ ^135^ ^]^ in 1989 and it consists in the selective observation of the interaction of bulk water protons with the protons contained in macromolecules of a tissue. **Figure** **10** schematizes the MTC process.

Two water pools are present in tissue (Figure 10A), namely, free bulk water and water molecules bound to macromolecules.^[^ ^70^, ^117^, ^135^ ^]^ The two pools are in an exchange regime. By irradiating the NMR resonances of protons belonging to immobilized, semisolid macromolecules, there is a direct saturation of bound water resonance. As a consequence of the chemical exchange between the two water pools, bulk water resonance is indirectly saturated. This generates contrast in the MR image. Representative Z‐spectrum of tissue (Figure 10B) shows the presence of the two water components, i.e., free water (long *T*_2_) with a narrow dip in the Z‐spectrum (few ppm), and the bound water (short *T*_2_) with a broad dip (hundreds ppm).

Both signals are cantered at the same frequency (0 ppm). In an MTC experiment, the rf saturation field is placed at a chemical shift far from bulk water resonance (Figure 10C). Since different tissues have different macromolecular compositions, the MTC can generate tissue contrast, based on physiochemical properties (Figure 10D).

It has been demonstrated that the detection of GBCAs by looking at its effect on MTC signal is more sensitive than using conventional *T*_1w_‐MRI. Our group applied this methodology for two different aims in which the detection threshold of GBCAs was challenging. The first application was for the imaging of Gd(III)‐labeled cancer cells in vivo. We demonstrated that MTC allowed detecting in vivo samples containing only 2% of Gd(III)‐labeled cells diluted in unlabeled cells, forming tumor mass. Conventional *T*_1w_‐MRI required a concentration of labeled cells ≈5 times higher.^[^ ^134^ ^]^

The second application was for the in vivo targeting of tumor cells and their visualization by MRI, a crucial challenge in biomedicine. For this aim, we developed cyclic‐RGD‐functionalized giant unilamellar vesicles (GUVs) loaded with Gd(III) complexes, able to accumulate at the cancer region. The presence of GBCA was indirectly assessed by exploiting MTC. The use of MTC opened the possibility of detecting Gd(III)‐GUVs, whereas not feasible by using conventional *T*_1w_‐MRI.^[^ ^136^ ^]^

In analogy, the effect of GBCAs can be indirectly assessed by looking at CEST signals of molecules endogenously present in the regions in which the GBCA distribute. Examples of endogenous CEST signals can be the amide proton transfer (APT) or the CEST_@2ppm_.^[^ ^117^, ^122^, ^137^ ^]^ The APT signal is generated from amide protons in endogenous proteins and peptides, in chemical exchange with bulk water protons. Its effect is very important in tissues and especially in cancer, where the content of mobile proteins and peptides is high (i.e., increased APT signal). The APT signal resonated at ≈3.4 ppm from bulk water proton signal. CEST_@2_ _ppm_ signal is generated by creatine, creatinine, and other amine‐containing endogenous molecules whose CEST resonance is centered at ≈2 ppm from bulk water proton signal.^[^ ^122^, ^137^ ^]^ In a CEST experiment, the observed saturation transfer (ST%) is a function of several parameters, namely, proton exchange rate, concentration of the pool of exchanging protons, intensity of the applied rf pulse, and the *T*_1_ of the bulk water protons, accordingly to the following equation

(1) $$\mathrm{ST}\;=1-\frac{I_{s}}{I_{0}}=\frac{k_{\mathrm{ex}\;}f_{\mathrm{CEST}}}{R_{1}^{W}+k_{\mathrm{ex}}f_{\mathrm{CEST}}}\left(1-e^{-t_{\mathrm{sat}\;}\left(R_{1}^{W}+k_{\mathrm{ex}}f_{\mathrm{CEST}}\right)}\right)$$

*f*_CEST_ is the molar fraction of the CEST protons, i.e.,

(2) $$f_{\mathrm{CEST}}=\frac{n\left[\mathrm{CA}\right]}{2\left[\mathrm{bulkW}\right]}$$

where [CA] is the concentration of the CEST agent and [bulkW] is the concentration of the bulk water (i.e., ≈55 mol L^−1^).

In the presence of a paramagnetic GBCAs in the extracellular region, the *T*_1_ of the bulk water protons is affected by the relaxivity and concentration of the GBCAs. Therefore, the presence of GBCAs (*T*_1_ shortening) can be indirectly detected by looking at CEST signal of molecules which are distributed in the same region.

Both detecting GBCAs by looking at their indirect effect on MTC or on CEST signals can be interesting in view of reducing the dose of administered GBCAs, since the techniques revealed to be more sensitive than conventional *T*_1w_‐MRI.^[^ ^117^, ^134^, ^136^, ^137^ ^]^

### 4.3 Recovery and Recycling

We discussed above the implications of the use of GBCAs, in terms of their tissue retention and the environmental contaminants. Thus, it is essential to reduce their use (e.g., by increasing the obtainable contrast) and to recover them after renal excretion due to their stability. Tracking anthropogenic Gd(III) helps monitor contaminant diffusion from densely populated areas or Gd(III)‐based MRI facilities. Efforts should focus on developing metal‐free CAs to avoid environmental metal recovery. Reducing the required concentration of agents or developing new protocols and instruments to increase sensitivity is crucial. Significant efforts are directed toward studying targeting agents or new adducts.

**Figure 11:**
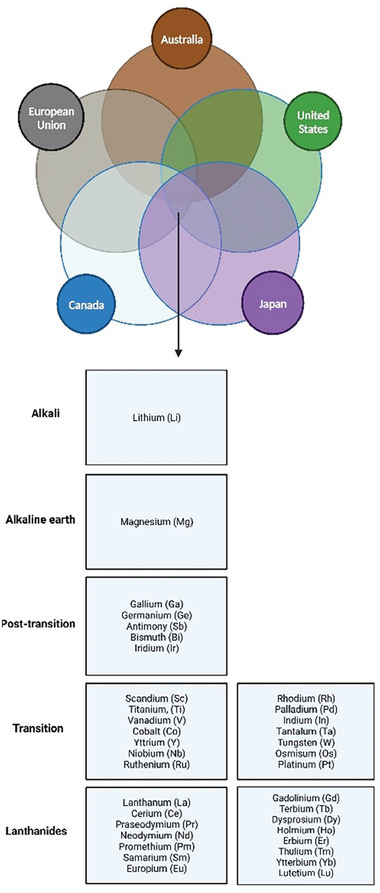
Determination of which metals is critical for recovery from water. A Venn diagram showing the common critical metals designated by five governing bodies (Austria, Canada, European Union, United States). Metals present in the list are considered critical for recovery by all the five governing bodies.

The costs and time required to translate new technologies and molecules from bench to bedside are still a concern, given the rising consumption of CAs. Recycling lanthanides could reduce extraction and environmental contamination, as noted by Schmidt et al. in 2019.^[^ ^64^ ^]^ Although the free ions cause significant issues recycling complexes require careful consideration. Ion chelators that release ions should be studied with priority. Excreted complex, through the urine, reaches the wastewater in a form and a quantity, which is quite the same as the injection, due the prolonged half‐life of the complexes. The presence of Gd(III) in the free form could be a consequence of the so‐called transmetalation processes: the lanthanides atom is substituted by other lanthanides or metal ions (or endogenous cations as Fe(III), Zn(II), Cu(II), and Ca(II)). The traces of the complexes in wastewater show the inefficacy of the filtering systems already available for the sequestering of this class of chemicals. Specific studies are needed to trace the spread of contaminants and the potential risks affecting biological systems, with a focus on human beings, in all the stages of development, and for all the concentrations intended to be used in clinics. While the focus of this review is mainly on the Gd(III) as a contaminant from biomedical applications, DuChanois et al.^[^ ^138^ ^]^ tried to identify, which are the critical metals worth to be recovered from wastewater and brines, taking into account three factors: the percentage of metals obtained as a companion, the functional end‐of‐use recycling rate, and how critical each metal is considered from five governing bodies (**Figure** **11**).

**Figure 12:**
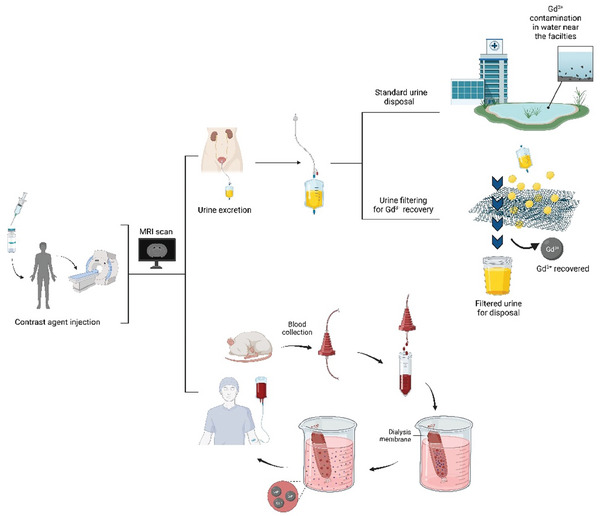
Schematic representation of possible roots of recovery of gadolinium from patients.

Of interest could be a method based on functionalized silica sorbent, as proposed by Ngamcherdtrakul et al.,^[^ ^139^ ^]^ for the removal of gadodiamide from the blood, to decrease the risk of NSF occurrence, with both an in vivo and ex vivo hemoperfusion approach (**Figure** **12**).

Also Yantasee et al.^[^ ^140^ ^]^ selective removal of lanthanides from natural waters, acidic streams, and dialysate) tested the binding affinity of lanthanides (La, Ce, Pr, Nd, Eu, Gd, and Lu) on self‐assembled monolayers on mesoporous silica supports (SAMMS) easily prepared and functionalized in their termination. The *K*_d_ distribution coefficient was assessed in acidic solutions, in natural waters, and in dialysate to test all the possible sources for lanthanides recovery with major attention on SAMMS regeneration after the use.

Pointing out how poor literature is in this field, another interesting approach is the one proposed by Zanardo et al., on the reduction of CA from hospital wastewater.^[^ ^141^ ^]^ In the GREENWATER study, they proposed the first step of a longer process, the quantification of GBCAs retrievable from the urines of the patients, collected after MRI or CT (computed tomography) exams.

**Figure 13:**
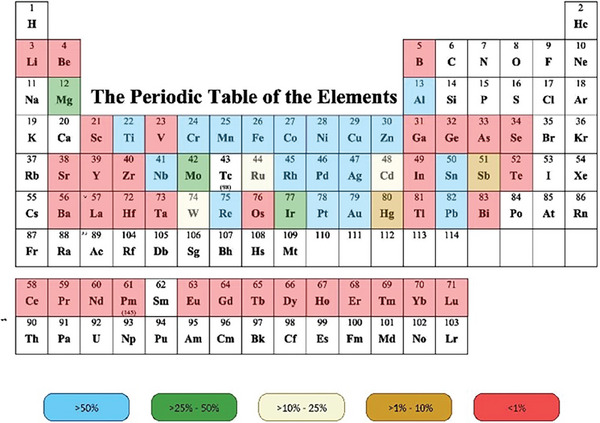
Substance specific recycling. Data are divided into five bins: >50%, 25–50%, 10–25%, 1–10%, and <1%.

The urgent need for tools for the selective removal of lanthanide atoms is not only strictly related to Gd(III), but also to the complete series of lanthanides and involves the REEs (**Figure** **13**).

The classical methods of solvent extraction and chromatography are not easily applicable for a scale‐up, which is the reason why the sorption is considered the use of selective mesoporous silica, zeolites, activated carbon, and metal–organic frameworks (MOFs).

An interesting solution was proposed by Sadeghi Chevinli et al.^[^ ^142^ ^]^ They proposed the synthesis of microporous Ce‐BTC (1,3,5‐benzenetricarboxylic acid) MOF, for the removal of La(III) from aqueous solutions due to the high porosity, easiness of preparation and the adjustable pore size. The idea of a sharable platform for the sequester of the full series of lanthanide atoms should be considered. New filtering systems must be biocompatible, able to collect contaminants and their degradation, and at the same time filters should be easy to regenerate, providing several life cycles.

**Figure 14:**
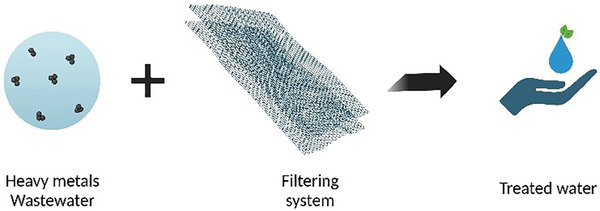
Schematic GO filtering system.

Following this rationale, graphene oxide (GO) has gained a lot of attention as an emerging efficient, economically affordable material that is capable of binding and retaining heavy metals from water (**Figure** **14**).^[^ ^140^, ^143^ ^]^

Activated carbon and graphene oxide have arisen as promising materials for metal recovery and removal from polluted aqueous matrices and industrial discharges.

Activated carbon is a highly porous material with a large surface area, obtained through processes like carbonization and activation of organic materials. This increased surface area provides numerous adsorption sites for heavy metal ions to attach to, effectively removing them from water systems. The adsorption mechanism is primarily owing to van der Waals forces, electrostatic interactions, and chemical bonding. Activated carbon can be tailored to target specific heavy metal ions by modifying its surface chemistry, allowing for efficient and selective removal.

The recovery of heavy metals using these materials typically involves mixing the adsorbents with the contaminated water. The adsorption process is influenced by factors like pH, contact time, initial concentration of heavy metals, and temperature. One significant advantage of using activated carbon and graphene oxide for heavy metal recovery is their potential for regeneration and reuse. After adsorption, the loaded adsorbents can undergo desorption processes to release the accumulated heavy metal ions. This regenerates the adsorbent material for subsequent cycles of use, reducing waste and costs associated with disposal.

Furthermore, finding a way to recover metals still in complex form is intriguing and useful. Elizalde‐González et al.^[^ ^144^ ^]^ proposed three different activated carbon samples and three GBCAs to study the adsorption by the means of urine models, illustrating the necessity for a more comprehensive investigation of the subject, including functional groups on the carbon surface, solution pH, and competitive adsorption processes.

GO is a 2D material composed of a single layer of carbon atoms arranged in a hexagonal lattice. It possesses exceptional mechanical, thermal, and electrical properties. The oxygen‐containing functional groups on its surface make it highly hydrophilic and reactive, making GO a great candidate for metal adsorption and a unique platform for conducting numerous reactions.^[^ ^140^ ^]^ The oxygen groups provide binding sites for heavy metal ions, enabling efficient adsorption through processes like electrostatic attraction, ion exchange, and coordination bonding.

In conclusion, both activated carbon and graphene oxide show great promise in heavy metal recovery due to their high adsorption capacities, selectivity, and potential for regeneration. Continued research and development in this field can lead to more efficient and sustainable solutions for addressing heavy metal pollution in various environmental settings.

## 5 Summary and Outlook

In conclusion, this review highlights the critical importance of reducing the dose of GBCAs administered to patients as MRI contrast agents, as well as the urgent need to minimize environmental contamination associated with their usage. Hence, a 4′R proposal must be strongly considered, consisting in i) Replacing (if possible) GBCA with other MRI CAs (e.g., Mn‐ or Fe‐based CA or diaCEST agents), ii) Reducing the dose of GBCAs (with improved MRI sequences/data analysis or improved relaxivity), iii) Recovering Gd(III) from the environment and Recycling it in view of circular economy and environmental sustainability.

The use of GBCAs has revolutionized the field of medical imaging, enabling enhanced visualization and improved diagnosis of various diseases. However, more recently important concerns regarding the potential adverse effects associated with Gd(III) deposition in the body (Gd(III) accumulation and retention) and its release into the environment have emerged.

Consequently, it is crucial to adopt strategies that optimize the administration of GBCAs, tailoring the dosage while ensuring the lowest effective concentration.

Furthermore, the environmental impact of Gd(III) release cannot be ignored. The accumulation of Gd(III) in aquatic systems, primarily through the discharge of contrast agent residues, poses a significant threat to ecosystems. It is imperative to develop and implement stringent protocols for the proper disposal and treatment of Gd(III)‐contaminated waste to prevent long‐term environmental consequences.

To address these concerns, future research efforts should focus on i) the design of methods to detect lower doses of GBCAs, as an alternative to conventional clinical MRI sequences and ii) developing methods to recover Gd(III) while minimizing environmental contamination.

To conclude, to mitigate environmental pollution caused by Gd(III), two complementary approaches are needed: reducing the injected contrast dose before scans and recovering the agent afterward. By prioritizing patient safety and environmental stewardship, the field of MRI contrast agents can continue to advance while minimizing potential harm to both individuals and ecosystems, thereby environmental sustainability (One Planet, One Health).

## Conflict of Interest

The authors declare no conflict of interest.
